# Targeted systemic dendrimer delivery of CSF‐1R inhibitor to tumor‐associated macrophages improves outcomes in orthotopic glioblastoma

**DOI:** 10.1002/btm2.10205

**Published:** 2020-12-11

**Authors:** Kevin Liaw, Rajsekhar Reddy, Anjali Sharma, Jiangyu Li, Michelle Chang, Rishi Sharma, Sebastian Salazar, Sujatha Kannan, Rangaramanujam M. Kannan

**Affiliations:** ^1^ Department of Chemical and Biomolecular Engineering Johns Hopkins University Baltimore Maryland USA; ^2^ Center for Nanomedicine, Department of Ophthalmology Johns Hopkins Medicine Baltimore Maryland USA; ^3^ Department of Biomedical Engineering Johns Hopkins University Baltimore Maryland USA; ^4^ Anesthesiology and Critical Care Medicine Johns Hopkins Medicine Baltimore Maryland USA

**Keywords:** dendrimer, glioblastoma, immunotherapy, systemic, targeted delivery, tumor‐associated macrophage

## Abstract

Glioblastoma is the most common and aggressive form of primary brain cancer, with median survival of 16–20 months and a 5‐year survival rates of <5%. Recent advances in immunotherapies have shown that addressing the tumor immune profile by targeting the colony‐stimulating factor 1 (CSF‐1) signaling pathway of tumor‐associated macrophages (TAMs) has the potential to improve glioblastoma therapy. However, such therapies have shown limited successes in clinical translation partially due to lack of specific cell targeting in solid tumors and systemic toxicity. In this study, we present a novel hydroxyl dendrimer‐mediated immunotherapy to deliver CSF‐1R inhibitor BLZ945 (D‐BLZ) from systemic administration selectively to TAMs in glioblastoma brain tumors to repolarize the tumor immune environment in a localized manner. We show that conjugation of BLZ945 to dendrimers enables sustained release in intracellular and intratumor conditions. We demonstrate that a single systemic dose of D‐BLZ targeted to TAMs decreases pro‐tumor expression in TAMs and promotes cytotoxic T cell infiltration, resulting in prolonged survival and ameliorated disease burden compared to free BLZ945. Our results demonstrate that dendrimer‐drug conjugates can facilitate specific, localized manipulation of tumor immune responses from systemic administration by delivering immunotherapies selectively to TAMs, thereby improving therapeutic efficacy while reducing off‐target effects.

## 1 INTRODUCTION

Glioblastoma multiforme is a highly aggressive and common brain cancer, representing ~55% of gliomas.^1^ Nearly 20,000 cases are diagnosed in the United States each year. The current standard of care involves maximum safe tumor resection with radiotherapy or chemotherapy. However, the median survival rate of patients with glioblastoma remains only 16–20 months and the 5‐year survival rate is less than 5%.^2^ In addition, patients face significant impacts to their cognitive functions and qualities of life.^3^ These prognoses have improved negligibly despite advancements in anticancer treatments,^4^, ^5^ thereby requiring the development of novel delivery strategies to address the significant technological gap between clinical needs and effective therapies.

Recent developments in cancer immunotherapies have revealed macrophage receptor signaling as a promising target due to their roles in regulating inflammatory responses.^6^ The activity of macrophages is governed by their cytokine expression profile, which can be broadly categorized into the classical M1 and alternative M2 phenotypes.^7^ To facilitate tumor progression, resident microglia and infiltrating macrophages are repolarized into an M2‐dominant phenotype through signals secreted by tumor cells.^8^ These tumor‐associated macrophages (TAMs) have been implicated in tumor initiation, progression, metastasis, and drug resistance in a variety of cancers.^9^, ^10^ Targeting TAMs to shift their polarization away from the tumor‐supporting state and toward an antitumor phenotype has become an attractive strategy in immunotherapies.

The colony‐stimulating factor 1 (CSF‐1) pathway has sparked interest to block tumor recruitment of macrophages into TAMs,^11^ and multiple clinical trials are underway for agents targeting the CSF‐1 pathway for cancer treatment (NCT02829723, NCT02452424, NCT01349049). CSF‐1 phosphorylation facilitates macrophage proliferation and conversion to TAMs, as well as tumor cell invasion and metastasis.^8^, ^12^ CSF‐1 receptor inhibitors such as BLZ945 has shown some promise for suppressing tumor progression in clinical studies. However, low response rates, poor brain and tumor penetration, and systemic safety concerns have been major obstacles limiting their clinical translation.^13^, ^14^ Therefore, targeted delivery vehicles that can bring immunotherapies from systemic circulation into brain tumors and specifically to TAMs while limiting off‐target activity may achieve significant improvements to glioblastoma treatment.

Hydroxyl‐terminated polyamidoamine (PAMAM) dendrimers are promising nanocarriers for delivering therapies specifically to activated microglia/macrophages from systemic circulation.^15^, ^16^ Their small size, neutral surface charge, and high density of surface hydroxyls enable them to cross the blood brain barrier and localize within activated microglia/macrophages without targeting moieties. This intrinsic targeting has been shown to significantly enhance the efficacy of antioxidants by targeting activated glia in neuroinflammation.^15^ In orthotopic brain tumor models, we have shown previously that systemically administered hydroxyl PAMAM dendrimers penetrate throughout the solid tumor and localize selectively in TAMs in an orthotopic model of malignant glioma.^17^, ^18^ These dendrimers are also well‐positioned for clinical translation due to their safe profile in vivo and scalability.^19^, ^20^ Here, we present a novel nanoparticle formulation of BLZ945 conjugated to the generation 4 hydroxyl PAMAM dendrimer via a triggered release linker that can locally repolarize the tumor immune profile for improved efficacy in glioblastoma treatment.

## 2 RESULTS

### 2.1 Preparation and characterization of dendrimer‐BLZ945 conjugate

**FIGURE 1:**
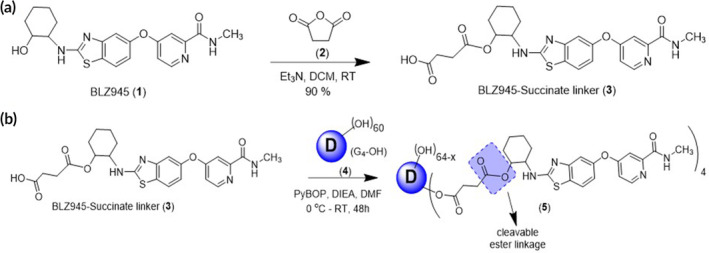
Schematic representation of the preparation of (a) BLZ945‐succinate linker (3) and (b) esterase cleavable dendrimer‐BLZ945 conjugate (5)

**FIGURE 2:**
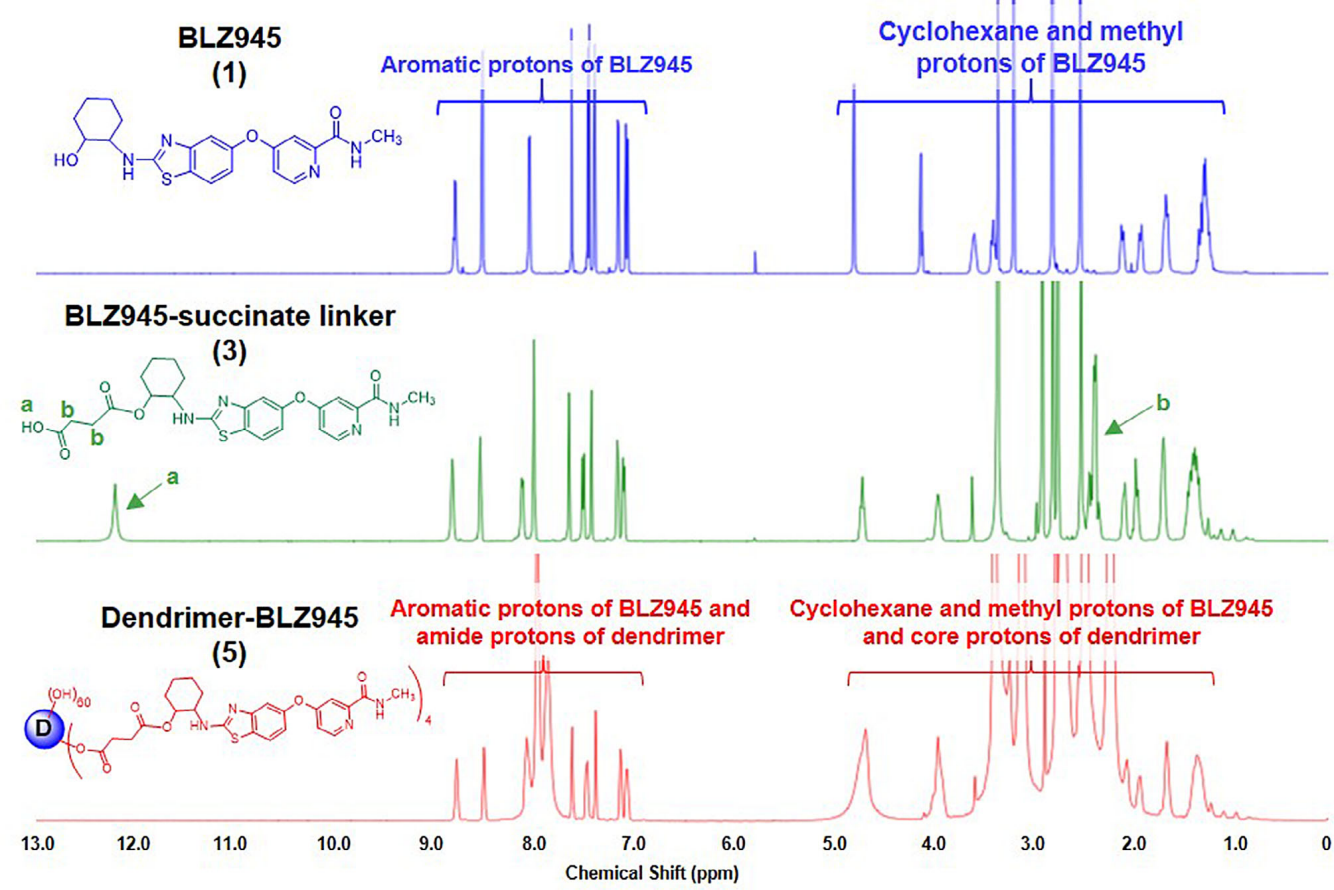
^1^H NMR characterization of BLZ945 (1), BLZ945‐succinate linker (3), and dendrimer‐BLZ945 conjugate (5). ^1^H‐NMR spectra of compounds indicate successful formation of (3) and successful chemical conjugation of (3) to (5). Key identifying protons are denoted

To improve formulation and delivery, we conjugated BLZ945 (BLZ) (**1**) to G4‐OH dendrimers. The hydroxyl group on the cyclohexane ring in BLZ945(**1**) was modified by reacting with succinic anhydride (**2**) to create BLZ945 succinate linker (**3**) with an enzyme cleavable linkage and a carboxylic acid terminal group for covalent attachment to the dendrimer surface (Figure 1a). Formation of the BLZ945‐succinate linker (**3**) was confirmed by ^1^H‐NMR (Figure 2). BLZ945‐succinate linker (**3**) exhibits peaks at 12.1 ppm corresponding to the carboxylic acid proton (a) and at 2.3 ppm corresponding to methylene protons on the linker (b).

**FIGURE 3:**
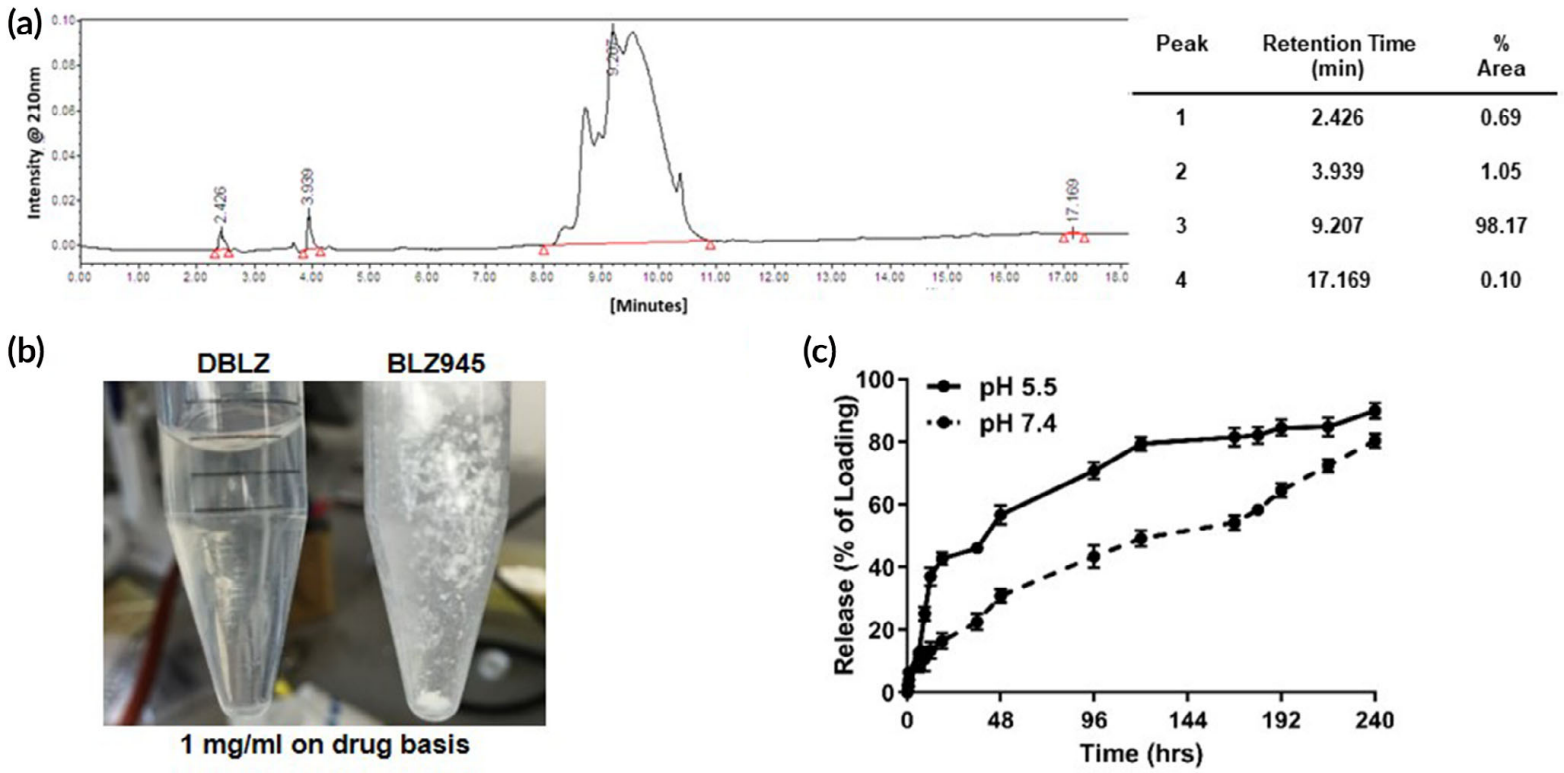
Characterization of dendrimer‐BLZ945 conjugates. (a) HPLC chromatogram confirming the purity of DBLZ as >98%. (b) Conjugation of BLZ945 to the dendrimer significantly improves its aqueous solubility. (c) DBLZ provides sustained pH sensitive drug release. BLZ945 is released from the conjugate more rapidly at pH 5.5 (lysosomal, solid) compared to pH 7.4 (plasma, dotted) conditions, with 43% released after 24 h versus 16%, respectively. Error bars denote mean ± SE. Each point represents *N* = 3 independent experiments with *n* = 2 internal replicates each. HPLC, high performance liquid chromatography

BLZ945‐succinate linker (**3**) was then conjugated directly to the dendrimer surface (**4**) through the formation of an ester linkage (Figure 1b). The dendrimer‐BLZ945 conjugate (DBLZ, **5**) was designed for pH and esterase sensitive triggered drug release in intracellular or intratumor conditions.^21^, ^22^ Formation of DBLZ (**5**) was confirmed with ^1^H‐NMR showing the presence of both BLZ (**1**) and dendrimer (**4**) protons, as well as the disappearance of the linker carboxylic acid proton (a) at 12.1 ppm after ester bond formation (Figure 2). DBLZ exhibited a molecular weight of ~16,200 Da, hydrodynamic diameter of 5.3 nm, and a ζ‐potential of +2.5 ± 0.1 mV. ^1^H‐NMR peak integration indicated a drug loading of 3–4 molecules of BLZ945 per dendrimer for 8–10 wt%/wt% per dendrimer. We have shown that the intrinsic macrophage targeting of dendrimers is preserved up to at least 20 wt/wt% drug loading.^23^ This 8–10 wt/wt% loading was chosen to account for this as well as the poor aqueous solubility of BLZ. The purity of the final conjugate was greater than 98% as verified using high performance liquid chromatography (HPLC) (Figure 3a). Conjugation to the dendrimer significantly increased the solubility of BLZ >100‐fold (Figure 3b), facilitating translation by removing the need for solvents or excipients in the formulation.

DBLZ demonstrated pH‐sensitive release of BLZ (Figure 3c). HPLC analysis confirmed BLZ was released as free drug rather than BLZ945‐succinate linker. In acidic pH simulating intracellular or intratumor conditions, DBLZ released 42.7% of the BLZ loading after 18 h compared to 16.3% in pH 7.4 conditions. In addition, DBLZ exhibited sustained drug release, with BLZ continually released from the dendrimer over 10 days. Taken together, the dendrimer TAMs targeting and triggered release of BLZ indicates that DBLZ may yield a promising therapeutic window for effective and safe glioblastoma treatment.

### 2.2 Dendrimer‐BLZ945 conjugate reduces CSF‐1 receptor phosphorylation and anti‐inflammatory expression in BV2 microglial cells

**FIGURE 4:**
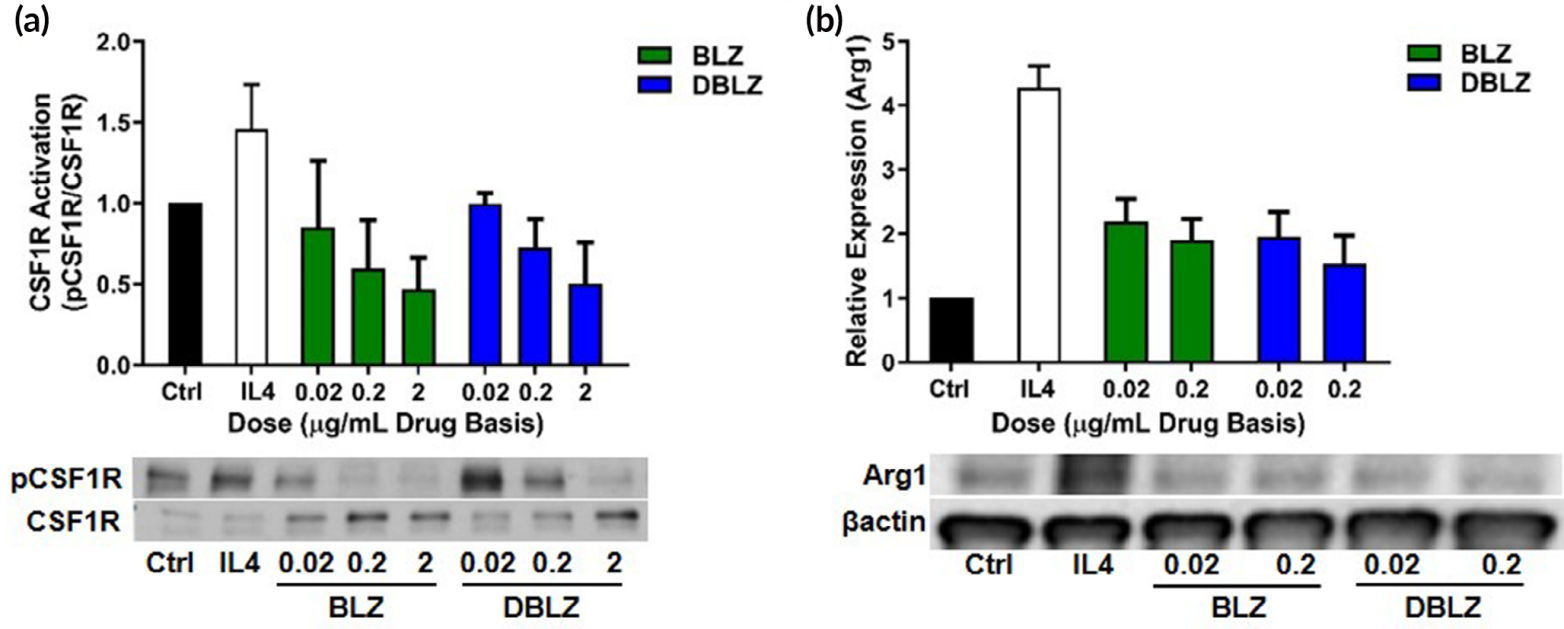
Treatment with BLZ945 (BLZ) or dendrimer‐BLZ945 conjugate (DBLZ) inhibits CSF‐1 receptor activation and arginase‐1 (Arg1) expression in IL4 stimulated microglia. BV2 murine microglia were treated with BLZ or DBLZ for 1 h, followed by cotreatment with IL4 at 100 ng/ml for 24 h. (a) Treatment with both BLZ and DBLZ inhibited IL4 induced CSF‐1 receptor phosphorylation in a dose‐dependent manner. (b) Both treatments also reduced IL4 induced Arg1 expression, a marker of pro‐tumor polarization. Error bars denote mean ± SE. Each bar represents *N* = 3 independent experiments with *n* = 3 internal replicates each. CSF‐1, colony‐stimulating factor 1; IL4, interleukin‐4

Neither BLZ nor DBLZ exhibited cytotoxic effects in the concentration range used for in vitro studies (Figure S3). There have been conflicting reports as to whether the therapeutic action of BLZ945 arises from ablation or repolarization of TAMs.^24^, ^25^ To evaluate the CSF‐1 receptor inhibitory properties of DBLZ compared to BLZ, treatments were assessed in vitro in BV2 microglia stimulated with interleukin‐4 (IL4), a TAMs activator that correlates with tumor prognoses.^26^ Stimulation with IL4 increased CSF‐1 receptor phosphorylation ~1.5‐fold, while treatment with both BLZ and DBLZ demonstrated dose‐dependent knockdown of CSF‐1 receptor activation (Figure 4a). Treatment at 0.02 μg/ml drug dose with both BLZ and DBLZ reduced phosphorylation to control levels, while treatment with both at 2 μg/ml reduced phosphorylation by ~50% compared to unstimulated controls. This knockdown in receptor activation translated to dose‐dependent decrease in expression of arginase‐1 (Arg‐1), an anti‐inflammatory cytokine that mediates tumor progression and metastasis and correlates with cancer grade and prognosis (Figure 4b).^27^ Notably, during the timeframe of in vitro experiments (24 h), only ~50% of BLZ is released from the dendrimer surface. Consistent with previous studies, this suggests that dendrimers exhibit high levels of cellular internalization to achieve similar efficacy as free BLZ despite only half the BLZ loading being released.^28^ We hypothesize based on the similar activity in vitro of BLZ and DBLZ, dendrimer transport and TAMs targeting by DBLZ in vivo will translate to improved effective dosage within TAMs for improved immune reprogramming.

### 2.3 Dendrimer delivery improves BLZ945 repolarization of the tumor immune environment

To evaluate effects on the tumor immune environment, orthotopic glioblastoma tumor bearing mice were injected intravenously via tail vein injection with a single dose of 100 mg/kg BLZ or DBLZ on day 10 after tumor inoculation and analyzed 4 days after treatment. Delayed systemic treatment was chosen despite the challenging administration route and treatment time to mimic clinical delays in tumor diagnosis, where glioblastoma exhibits well‐developed solid tumors and potential multiple lesions by the time of diagnosis and treatment initiation.^29^

**FIGURE 5:**
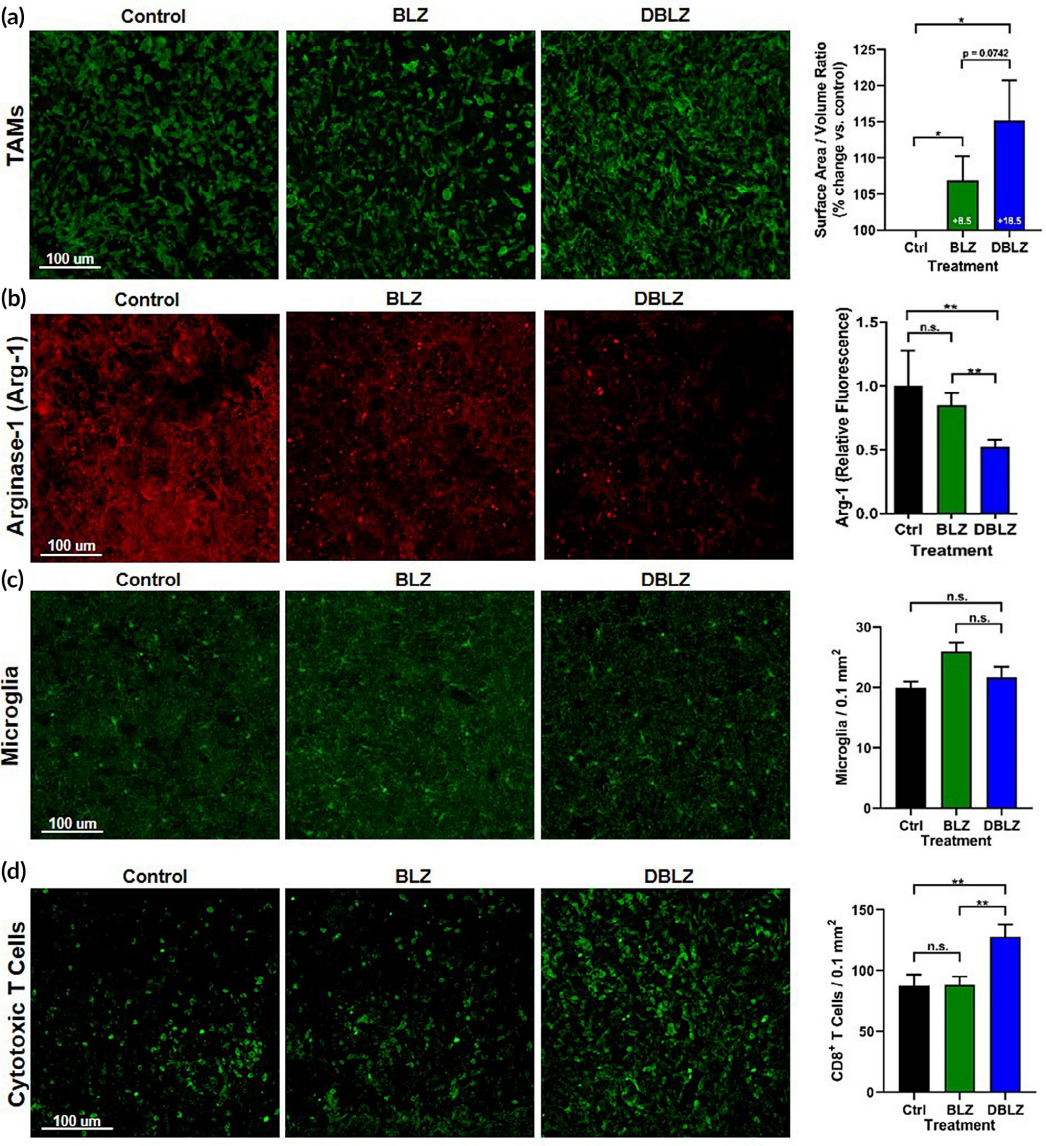
Targeted delivery of BLZ945 (BLZ) with dendrimers (DBLZ) enables phenotype switching of tumor‐associated macrophages (TAMs) from pro‐ to anti‐tumor polarization. Glioblastoma tumor‐bearing mice were treated with one dose of 100 mg/kg BLZ or DBLZ on day 10 after inoculation. Brains were collected 4 days after treatment, fixed, and stained for confocal imaging. (a) DBLZ treatment increases the surface area to volume ratio of tumor‐associated macrophages (TAMs, green), indicating reduced pro‐tumor macrophage polarization. *p < .05. (b) Expression of arginase‐1 (Arg1), a pro‐tumor cytokine, within TAMs is significantly reduced with DBLZ treatment whereas BLZ treatment demonstrates a lesser effect. **p < .01, n.s. *p* > .1. (c) DBLZ treatment showed no change in microglia presence in the contralateral hemisphere compared to control and BLZ treatment groups. n.s. *p* > .1. (d) DBLZ treatment increases the presence of cytotoxic CD8+ T cells (green) within the tumor, indicating strengthened anti‐tumor immune response. *p < .05, n.s. *p* > .1. Error bars denote mean ± SE. Each bar represents *N* = 10 animals with *n* = 3 images analyzed for each

TAMs in the tumor exhibited highly ameboid morphology, consistent with previous reports that TAMs exhibit loss of ramified structure correlating with immunosuppressive activation (Figure 5a).^17^ TAMs in mice treated with DBLZ exhibit partial recovery of ramified structure, while TAMs in free BLZ treated mice have ameboid morphology similar to TAMs in untreated mice. Surface area to volume ratio, a measure of sphericity, indicates that DBLZ treatment significantly increased this ratio ~2‐fold more (+18.5% vs. control; *p* = .07 BLZ vs. DBLZ) compared to free BLZ (+8.5% vs. control). We have observed previously that resting macrophages exhibit ~2‐fold greater surface area to volume ratio than TAMs, so DBLZ treatment induced a partial recovery away from TAMs activation and toward resting phenotype.^17^ Consistent with this, DBLZ significantly decreased the expression of Arg‐1 in TAMs compared to control (*p* = .005 control vs. DBLZ) and BLZ (*p* = .003 BLZ vs. DBLZ) treatment. Free BLZ showed no improvements compared to controls (Figure 5b). Interestingly, TAMs numbers within the tumor remained unchanged with BLZ or DBLZ treatment compared to controls (data not shown). In addition, healthy microglia in the contralateral hemisphere exhibited no change in numbers with DBLZ compared to control and BLZ treatments (Figure 5c, *p* = .43 control vs. DBLZ, *p* = .13 BLZ vs. DBLZ).

While manipulation of cytotoxic T cells has been the primary target since the advent of cancer immunotherapies,^30^ macrophage‐focused strategies have emerged that enable repolarization of the immunosuppressive tumor environment to indirectly promote antitumor activity of T cells. As mediators of the immune response, TAMs have been shown to impede the engagement of cytotoxic T cells correlating to the degree of CSF‐1 receptor activation.^31^ To explore the impact of tumor immune repolarization on downstream immune responses, tumors were analyzed for CD8^+^ cytotoxic T‐cell infiltration. DBLZ treatment significantly increased the presence of CD8^+^ cytotoxic T cells in the glioblastoma tumor (127.9 ± 17.4 CD8^+^ T cells per 0.1 mm^2^, *p* = .0013 control vs. DBLZ, *p* = .0026 BLZ vs. DBLZ) by ~50% compared to untreated (87.5 ± 15.6 T cells per 0.1 mm^2^) and BLZ945 (88.4 ± 11.5 T cells per 0.1 mm^2^) treated mice (Figure 5d), as a result of TAMs reprogramming.

### 2.4 Dendrimer delivery prolongs survival and improves behavioral markers of glioblastoma burden

To evaluate if improvements by DBLZ in reprogramming the tumor immune environment translated into improvements in overall outcomes, brain tumor bearing mice were injected intravenously via tail vein injection with a single dose of 100 mg/kg BLZ or DBLZ on day 10 postinoculation. Mice were observed for overall survival and recorded in open field experiments to assess motor and behavioral markers of disease progression.

**FIGURE 6:**
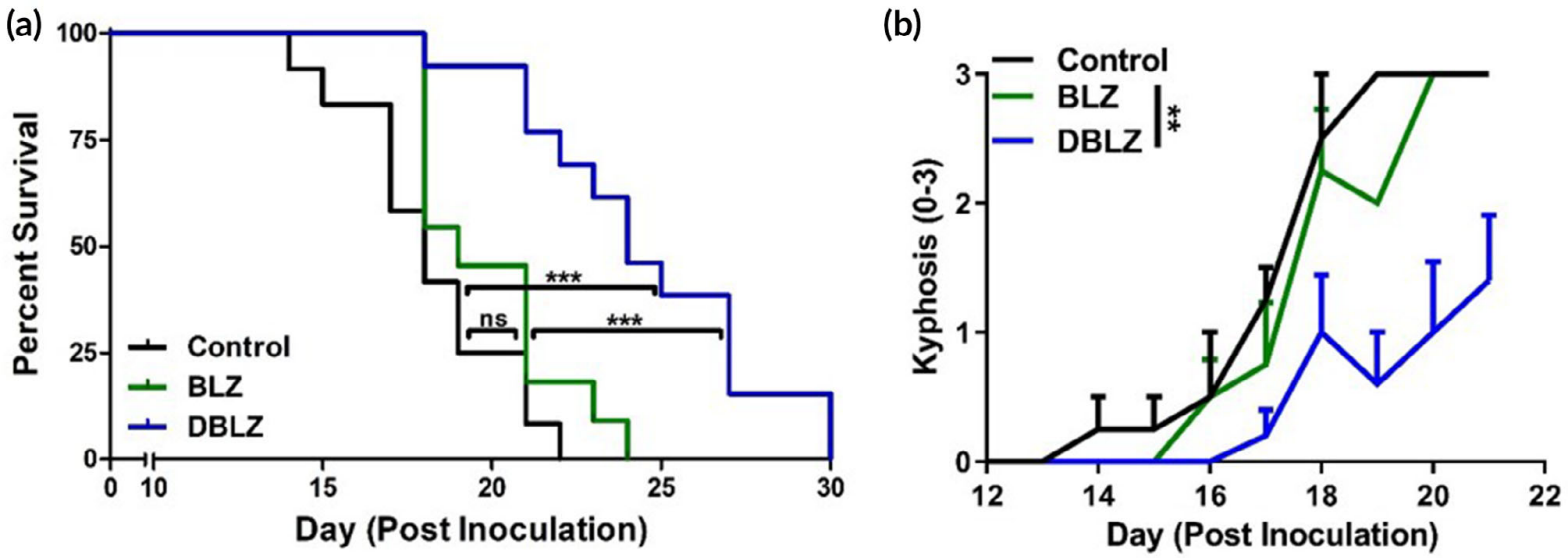
Treatment with dendrimer‐BLZ945 (DBLZ) improves outcomes in brain tumor‐bearing mice while BLZ945 (BLZ) treatment fails to demonstrate improvements to disease burden. C57BL/6 mice were inoculated with GL261 murine glioblastoma cells and treated systemically via intravenous administration with a single dose of 100 mg/kg BLZ or DBLZ on day 10 after inoculation. (a) A single systemic dose of DBLZ significantly prolonged survival outcomes in glioblastoma‐bearing mice compared to control and BLZ treated mice by ~30%. n.s. *p* > .1, ***p < .001. (b) DBLZ treatment significantly mitigated the onset and severity of kyphosis, a measure of disease burden, compared to control and BLZ treatments. **p < .01

**FIGURE 7:**
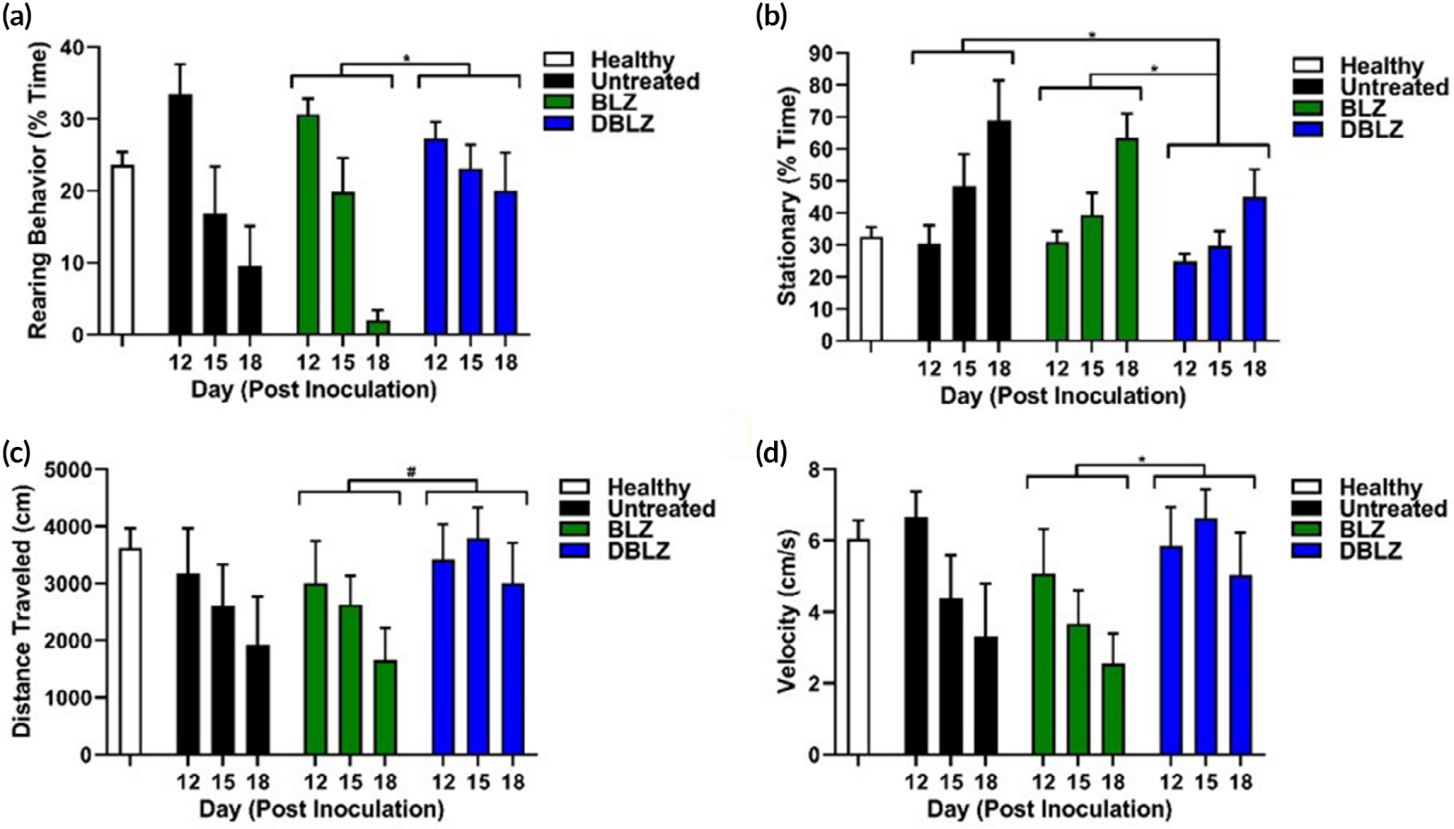
Treatment with dendrimer‐BLZ945 (DBLZ) improves markers of tumor burden in open field analyses. Open field experiments demonstrate that DBLZ treatment significantly improves (a) rearing behavior, (b) time spent stationary, (c) total distance traveled, and (d) velocity compared to untreated and free BLZ945 (BLZ) treated mice. These behavioral and motor markers are proxies for fatigue and anxiety, indicating that DBLZ improves these secondary markers of disease progression. *p < .05, ^#^ *p* < .1

A single systemic dose of DBLZ significantly improved overall survival compared to control and free BLZ (*p* < .0001 control vs. DBLZ, *p* = .0003 BLZ vs. DBLZ). BLZ exhibited no improvements over untreated mice (Figure 6a, *p* = .127 control vs. BLZ). Median survival time increased ~30% with DBLZ (24 days) treatment from untreated (18 days) and free BLZ (19 days) groups. In addition to prolonging survival, we also demonstrate that DBLZ treatment significantly improved markers of wellbeing in glioblastoma brain tumor bearing mice, while free BLZ exhibited minimal improvements compared to untreated mice. DBLZ treatment delayed the onset and mitigated the severity of kyphosis compared to BLZ and untreated groups (Figure 6b). Glioblastoma progression induced a deterioration in the rearing behavior of mice, while DBLZ significantly increased the exploratory time spent by mice compared to free BLZ treatment (Figure 7a, *p* = .023 BLZ vs. DBLZ). DBLZ treated mice also spent less time immobile (Figure 7b, *p* = .012 control vs. DBLZ, *p* = .023 BLZ vs. DBLZ). In terms of overall mobility, DBLZ treated mice exhibited greater total distance traveled (Figure 7c, *p* = .061 BLZ vs. DBLZ) and velocity (Figure 7d, *p* = .017 BLZ vs. DBLZ) compared to untreated and free BLZ treatment. Taken together, these results indicate that in addition to prolonging survival, a single systemically administered dose of DBLZ may also improve quality of life (QoL) and reduce drug toxicity.

## 3 DISCUSSION

Despite nanomolar binding affinity to the CSF‐1 receptor, BLZ requires high doses when administered systemically to achieve therapeutic efficacy (e.g., 200 mg/kg oral daily dose in orthotopic brain tumor models).^24^, ^32^ In addition, its low aqueous solubility and potential toxicity due to ablation of systemic macrophages and microglia in healthy brain tissue are major obstacles for its clinical translation.^13^, ^24^ To overcome these challenges, we conjugated BLZ to nontoxic hydroxyl PAMAM dendrimers undergoing clinical translation to achieve specific targeting to TAMs and improve aqueous solubility.^17^ We have previously demonstrated that these dendrimers penetrate into orthotopic brain tumors and localize specifically within TAMs, as well as in activated glia in central nervous system (CNS) disease models, with negligible uptake in other cell types.^16^, ^17^, ^18^ These dendrimers penetrate the blood brain barrier from systemic circulation and diffuse freely through brain tumor tissue; then only highly endocytic reactive inflammatory cell populations, such as TAMs, are able to internalize these dendrimers. Based on our previous work, we anticipate approximately 0.5% of the injected dose to reach the tumor and reside specifically within TAMs.^18^ The hydroxyl group on the cyclohexane ring in BLZ was chosen for modification with succinic acid linker for attachment to the dendrimer surface. This cyclohexanol is theorized to play a critical role in its CSF‐1 receptor inhibition for competitively binding to the receptor ATP binding site to block phosphorylation, and its modification has been shown to significantly reduce its inhibitory potency.^14^, ^33^ Therefore, by blocking the cyclohexanol group via conjugation of the hydroxyl group to succinic anhydride (**2**), we expect that BLZ will remain inactive while conjugated to the dendrimer to mitigate potential systemic toxicities until the dendrimer reaches TAMs for release. Triggered pH sensitive release was confirmed, with more rapid BLZ release from the dendrimer in acidic conditions compared to neutral conditions. Due to the observed adverse effects associated with BLZ, including ablation of healthy microglia in the brain and Kupffer cells in the liver,^13^, ^24^, ^34^ this triggered release will limit BLZ activity to the tumor microenvironment while remaining inactive in healthy tissues to reduce off‐target effects. In addition, the sustained release of BLZ from the dendrimer exhibited by DBLZ coupled with dendrimer residence within TAMs for at least 48 h after administration^17^ compares favorably to those of free anticancer drugs, which are cleared rapidly from the tumor.^35^, ^36^

CSF‐1 receptor inhibition as a method of achieving macrophage ablation has been explored as a therapeutic approach but has demonstrated inconsistent results.^24^, ^25^ We observed that TAMs counts within the tumor was not reduced with free BLZ or DBLZ treatment at these doses compared to controls. This is consistent with previous results where TAMs were shown to exhibit protection from CSF‐1 receptor inhibition depletion through glioma cell derived factors.^24^ However, other reports have shown that CSF‐1 receptor inhibition can induce apoptosis in TAMs ablation in other types of cancers.^37^ In these cases, significantly greater doses of CSF‐1 receptor inhibitors were used, potentially leading to the TAMs ablation. Further exploration is warranted into potential TAMs ablation with CSF‐1 receptor inhibition, glioma cell secretions that may confer TAMs survival, and how dendrimer delivery of CSF‐1 receptor inhibitors may interact with these factors. Notably, we also observed no change in the counts of healthy microglia in the contralateral hemisphere, in contrast to previous reports.^24^ This demonstrates that utilizing dendrimer TAMs targeting and triggered release, we are able to scale down the dosing requirement required to achieve efficacy, thereby reducing adverse impacts on resting microglia outside the brain tumor.

Previous studies in an orthotopic platelet‐derived growth factor‐driven glioma mouse model have shown that 200 mg/kg of daily systemic BLZ945 doses can significantly improve survival, with ~70% of BLZ945 treated mice surviving to 26 weeks, compared to a median survival time of ~6 weeks in untreated mice.^24^ Studies in cervical and mammary flank tumor models daily doses of 200 mg/kg have shown similar significant efficacy, with BLZ945 treatment reducing tumor burdens by ~10‐fold.^37^ Studies with BLZ945 and PLX3397, another CSF‐1 receptor inhibitor, have also found that these TAMs targeted strategies can be significantly improved through combination with chemotherapies or radiation.^38^, ^39^ These prior studies have focused on daily dosing of CSF‐1 receptor inhibitors at high doses starting immediately after tumor inoculation. Previous studies in nanoparticle‐mediated delivery of CSF‐1 receptor inhibitors have shown that such delivery may enable less frequent and lower dosing.^40^, ^41^ For these studies, to better reveal the differential efficacy between free BLZ and DBLZ treatments, we significantly scaled down the dosing scheme to a single systemic dose of 100 mg/kg administered halfway through the model disease progression. Notably, impacts on the tumor immune microenvironment achieved with this single dose of DBLZ compare favorably to these results in the literature despite exhibiting lesser efficacy in terms of overall survival. DBLZ reduced expression of Arg‐1 by ~50% with this single systemic dose, which compares favorably to previous studies of CSF‐1 receptor inhibition where ~70% in vivo knockdown of Arg‐1 expression in TAMs in orthotopic brain tumor models required daily repeated dosing of 40–100 mg/kg PLX3397.^39^, ^42^ Similarly, the significantly increased cytotoxic T cell infiltration was achieved with DBLZ with a single dose at significantly lower BLZ945 doses than these reports, demonstrating the advantages of selective targeted delivery of BLZ945 to TAMs by dendrimers. Notably, these advantages with DBLZ in TAMs repolarization and T cell infiltration were achieved at ~10‐ to 50‐fold lower doses compared to those studies. Based on these promising proof of concept studies, we have demonstrated that by using dendrimers to target BLZ945 to TAMs within orthotopic brain tumors to significantly increase the effective drug concentration at the site of therapeutic action, we can achieve significant improvements to survival and biomarkers of immune activations. Future studies will explore more aggressive dosing regimens and combination with other treatment modalities for synergistic efficacy.

Preclinical and clinical studies of anticancer medicines have long focused on survival and have used progression‐free survival as a proxy for QoL.^43^ However, recent patient surveys have revealed that survival and patient QoL are not well correlated, and studies of cancer therapies often underreport or underemphasize adverse impacts to patient wellbeing.^44^ Impacts of treatments on patient QoL is therefore an important, often overlooked parameter that should be taken into consideration, particularly in glioblastoma, which is associated with significant cognitive and behavioral burden.^3^ As proxies for QoL we assessed brain tumor bearing mice with parameters associated with mouse health and anxiety, including kyphosis, rearing behavior, and motor function. Kyphosis is a measure of hunched posture that is used as a marker for mouse health and in the context of neurodegeneration and glioblastoma results from spinal cord muscular atrophying.^45^, ^46^ Rearing behavior an exploratory instinct used in behavioral neuroscience as a marker for fear/anxiety associated with disease burden.^47^ Mobility in open field experiments as a proxy for anxiety and fatigue has been shown to correlate with tumor burden, with mice exhibiting decreased mobility when inoculated with tumors and partial recovery upon tumor resection.^48^, ^49^ Here we demonstrate that in addition to prolonging survival and reprogramming the tumor immune environment, a single systemically administered dose of DBLZ can significantly improve QoL measures in this model through specific BLZ delivery to brain tumor TAMs and systemic clearance intact via kidneys over ~48 h. Such measurements of patient wellbeing apart from overall survival are underexplored in preclinical anticancer studies and may provide a more wholistic evaluation of therapeutic efficacy.

## 4 MATERIALS AND METHODS

### 4.1 Materials

Generation 4 hydroxyl PAMAM dendrimers (G4‐OH) were obtained from Dendritech (Midland, MI). BLZ945 was obtained from ChemShuttle (Hayward, California). Succinic anhydride, dichloromethane (DCM), dimethyl formamide (DMF), triethylamine (TEA), methanol, *N,N′*‐diisopropylethylamine (DIEA), and bovine serum albumin (BSA) were obtained from Sigma Aldrich (St. Louis, Missouri). (Benzotriazol‐1‐yloxy)tripyrrolidino‐phosphonium hexafluorophosphate (PyBOP), 4‐dimethylaminopyridine, and *N,N′*‐dicyclohexylcarbodiimide were obtained from Bachem Americas, Inc. (Torrance, California). DMSO‐d6 was purchased from Cambridge Isotope Laboratories, Inc. (Andover, Massachusetts). Dialysis membranes were purchased from Repligen (Waltham, Massachusetts). Dulbecco's Modified Eagle's Medium (DMEM), RPMI, l‐glutamine, fetal bovine serum (FBS), penicillin‐streptomycin (P/S), 0.25% trypsin–EDTA, normal goat serum (NGS), anti‐rabbit Alexafluor 488 and 594 secondary antibodies, MTT reagent, and anti‐CD8 primary antibody reagent were purchased from Invitrogen (Carlsbad, California). T‐per lysis buffer, protease inhibitor, and phosphatase inhibitor were obtained from ThermoFisher (Waltham, Massachusetts). Murine CSF‐1 and interleukin 4 were obtained from R&D Systems (Minneapolis, Minnesota). Tris‐buffered saline (TBS), and phosphate‐buffered saline (PBS) were purchased from Corning (Corning, New York). Iba1 primary antibody was purchased from Wako Pure Chemical Corporation (Tokyo, Japan). Anti‐mouse arginase 1 (Arg‐1) primary antibody was purchased from Abcam (Cambridge, UK). NucBlue cell stain (4′,6‐diamidino‐2‐phenylindole, DAPI) was purchased from Cell Signaling (Danvers, Massachusetts).

### 4.2 Chemical characterization of compounds

Proton nuclear magnetic resonance (^1^H‐NMR) spectra were recorded on a Bruker 500 MHz spectrometer (Billerica, Massachusetts) at room temperature (RT). Chemical shifts are reported in parts per million (ppm) relative to tetramethylsilane internal standard.

HPLC was used to validate purity of compounds. The HPLC instrument was equipped with 1525 binary pump, 2998 photodiode array detectors, and 717 autosampler interfaced with Empower software (Waters Corporation, Milford, Massachusetts). Chromatograms were monitored at 254 nm. The mobile phase was a mixture of water and acetonitrile (ACN) (0.1 wt%/wt% trifluoroacetic acid, TFA) at a flow rate of 1 ml/min. The column used was a symmetry C18 column (300 A, 5 μm, 4.6 mm × 250 mm) with guard column. The gradient flow method used began at 90:10 water/ACN for 10 min, then to 10:90 water/ACN for 20 min, and back to the initial condition for 20 min. Reference HPLC traces for free BLZ945 and DBLZ are shown in Figures S1 and S2.

Dynamic light scattering and zeta (ζ) potential were measured using a Zetasizer Nano ZS from Malvern Instrument Ltd. (Worchester, UK) to determine particle size and surface charge. Dendrimers were prepared by dissolving in water at 0.1 mg/ml. Measurements were performed in at 25°C with a scattering angle of 173° according to previously published protocol.^50^

### 4.3 Synthetic protocol for DBLZ conjugate

#### 4.3.1 Synthesis of BLZ945 succinate linker (compound 3)

BLZ945 (1) (100 mg, 0.25 mmol) was dissolved in DCM (25 ml) under nitrogen atmosphere, to which TEA (210 μl, 1.5 mmol) was added and stirred for 10 min. Then succinic anhydride (2) (250 mg, 0.25 mmol) dissolved in DCM was added to the flask. The reaction was run for 24 h at RT. The completion of the reaction was monitored by thin layer chromatography (TLC). Upon completion, silica gel was added to form a slurry, and column chromatography was performed. The pure product was obtained in 5% methanol in DCM. The solution was evaporated on a rotary evaporator to yield BLZ945‐succinate linker (3) at 90% yield.

^1^H‐NMR (500 MHz, CDCl_3_) δ 8.41 (d, *J* = 5.5 Hz, 1H), 8.23 (s, 1H), 7.58 (d, *J* = 8.6 Hz, 1H), 7.37 (dd, *J* = 27.8, 2.3 Hz, 2H), 7.16 (dd, *J* = 5.5, 2.5 Hz, 1H), 7.01 (dd, *J* = 8.7, 2.3 Hz, 1H), 4.79 (td, *J* = 11.0, 4.5 Hz, 1H), 3.85 (s, 1H), 3.16–3.03 (m, 1H), 3.00 (d, *J* = 5.1 Hz, 3H), 2.43 (t, *J* = 8.3 Hz, 2H), 2.31–1.96 (m, 4H), 1.84 (d, *J* = 7.1 Hz, 2H), 1.62–1.29 (m, 4H).

#### 4.3.2 Synthesis of DBLZ (compound 5)

BLZ945‐succinate linker (120 mg, 0.24 mmol) was dissolved in DMF (20 ml). Then PyBOP (224.8 mg, 0.432 mmol) and DIEA (75 μl, 0.432 mmol) were added. The solution was stirred under nitrogen atmosphere in an ice bath for 20 min. Then G4‐OH (4) (336 mg, 0.024 mmol) was added to the mixture and stirred for 48 h at RT. The resulting solution was then diluted with DMF and dialyzed against DMF (2 kDa MWCO) for 48 h with frequent solvent changes. The solution was further dialyzed against water and the aqueous solution was lyophilized to obtain a fluffy light‐yellow solid powder of pure DBLZ.

^1^H‐NMR (500 MHz, DMSO) δ 8.76 (d, *J* = 4.6 Hz, BLZ945 H), 8.48 (d, *J* = 4.6 Hz, BLZ945 H), 8.11–7.75 (m, dendrimer internal amide), 7.59 (d, *J* = 18.8 Hz, BLZ), 7.46 (d, *J* = 8.7 Hz, BLZ), 7.37 (s, BLZ), 7.12 (s, BLZ), 7.06 (d, *J* = 8.6 Hz, BLZ), 4.72 (bs, dendrimer OH), 4.02–3.80 (m, BLZ and dendrimer H), 3.44–3.23 (m, dendrimer backbone), 3.19–2.96 (m, dendrimer backbone and linker), 2.63–2.55 (m, dendrimer backbone), 2.40–2.33 (m, dendrimer backbone), 2.29–2.11 (m, dendrimer backbone), 1.71–1.59 (m, BLZ), 1.45–1.21 (m, BLZ).

### 4.4 BLZ945 release from dendrimer conjugate

The drug release of BLZ945 from the DBLZ were measured in PBS (pH 7.4) and citrate buffer (pH 5.5) to simulate plasma and intracellular/intratumor conditions, respectively, based on previously published procedure.^51^ 3 mg/ml DBLZ solutions (10 ml; *n* = 3) for each condition was maintained at 37°C on a shaker table. DBLZ was directly dissolved in the release mediums. The aliquots were taken out at appropriate time‐points. Samples were flash frozen to halt drug release. BLZ945 release was quantified using HPLC by analyzing the free BLZ945 peak in release samples compared to BLZ945 calibration curves.

### 4.5 Cell culture

BV2 murine microglia were obtained from Children's Hospital of Michigan Cell Culture Facility. Authentication and *Mycoplasma* testing were not performed. Cells were cultured in DMEM with 10% FBS and 1% P/S at 37°C and 5% CO_2_. Treatments were in DMEM with 5% FBS and 1% P/S. Cytotoxicity of BLZ and DBLZ were determined by measuring cell viability after 24 h of exposure via MTT assay. Cells used were thawed and used within 1–2 passages for experiments.

### 4.6 In vitro analysis of CSF‐1 receptor activation and Arg‐1 expression by Western blot

To measure CSF‐1 receptor activation (phosphorylation) and Arg‐1 expression, cells were treated with BLZ or DBLZ for 1 h followed by cotreatment with IL‐4 at 50 ng/ml for 24 h and collected in t‐per buffer supplemented with protease inhibitor and phosphatase inhibitor. Treatments were applied prior to IL‐4 stimulation to simulate the blocking of macrophage recruitment and polarization by tumor‐secreted signals, where the drug activity occurs prior to cell interaction with the stimulating cytokine.

Cells were lysed in t‐per buffer by high speed vortexing for 30 s and incubating on ice for 5 min. Samples were centrifuged down to collect the cell protein solution. Protein concentrations were determined using a BCA protein assay (ThermoFisher). Protein samples were separated on 4–15% gradient sodium dodecyl sulfate polyacrylamide gel electrophoresis (SDS‐PAGE gels, Bio‐Rad Laboratories, Hercules, California) at 120 V and transferred to polyvinylidene fluoride membranes (Pall Corporation, Port Washington, New York) at 80 V for 1 h. Membranes were blocked with 5 wt%/vol% BSA for 1 h at RT. Membranes were then incubated with primary antibodies overnight at 4°C, followed by incubation with horseradish peroxidase conjugated secondary antibody for 2 h at RT. Protein bands were visualized using chemiluminescence substrate (Promega, Madison, Wisconsin) and HyBlot CL autoradiography film (Denville Scientific, Inc., Metuchen, New Jersey) Quantitative densitometric analysis was performed using Multi‐Gauge software (Fujifilm, Tokyo, Japan).

The following primary antibodies were used: arginase‐1 (1:1000, Cell Signaling Technology, Danvers, Massachusetts), phosphorylated CSF‐1 receptor (1:1000), CSF‐1 receptor (1:1000), β‐actin (1:10,000, Sigma).

### 4.7 Tumor inoculations and treatment administration

All animals were housed at Johns Hopkins University animal facilities and were given free access to food and water. Experiments performed were approved by the Johns Hopkins Institutional Animal Care and Use Committee (MO16M122).

Male and female C57BL/6 mice 6–8 weeks of age were inoculated intracranially with GL261 murine glioblastoma cells obtained from the DTP/DCTD/NCI Tumor Repository (National Cancer Institute, Frederick, Maryland). GL261 were maintained in RPMI with 10% FBS, 1% P/S, and 1% l‐glutamine at 37°C and 5% CO_2_. Authentication and *Mycoplasma* testing were performed by the tumor repository. Cells were used within 2–3 passages after thawing. Mice were anesthetized with a ketamine (Vedco, St. Joseph, Missouri) and xylazine (Akorn Animal Health, Lake Forest, Illinois) cocktail. A midline scalp incision was made and a burr hole drilled 1 mm posterior to the bregma and 2 mm lateral to the midline. A 2 μl Hamilton syringe (Hamilton Company, Reno, Nevada) was lowered to a depth of 2.5 mm to inject 2 μl of GL261 solution containing 100,000 cells over 10 min using a stereotactic frame and automated syringe pump (Stoelting Co., Wood Dale, Illinois). The syringe was withdrawn at 0.5 mm/min, and the incision sutured.

### 4.8 Tissue processing, immunohistochemistry, confocal imaging, and image analysis for tumor immune environment

To assess the tumor immune environment, brain tumor bearing mice were treated with an intravenous dose of 100 mg/kg BLZ or DBLZ on day 10 after tumor inoculation via tail vein injection. Day 10 was chosen as the dosing time point because it corresponds to approximately halfway through disease progression in this model with the presence of a well‐developed tumor. Treatments were formulated in saline at equivalent BLZ dosage, with 5% DMSO in BLZ and control groups. Brains were collected on day 14 after tumor inoculation and fixed in 4% formalin solution overnight, followed by a daily sucrose gradient (10, 20, to 30% sucrose in PBS). Brains were then flash frozen. Brains were sectioned axially into 30 μm slices using a Leica CM 1905 cryostat (Wetzlar, Germany). Brains were then stained with DAPI to visualize cell nuclei and Iba1 to visualize TAMs or CD8 to visualize cytotoxic CD8^+^ T cells. Slices were blocked with 1xTBS + 0.1% Triton‐X + 1% BSA + 5% NGS for 4 h at RT. Then slices were incubated with primary antibodies (Iba1 1:200 or CD8 1:200) in 1xTBS + 0.1% Triton‐X + 1% BSA overnight at 4°C. Slices were then incubated with secondary antibody (goat anti‐rabbit 488 1:200) in 1x TBS + 0.1% Triton X for 2 h at RT. Finally, slices were incubated with DAPI for 15 min, mounted, and sealed. To stain for Arg‐1, a similar protocol was followed with anti‐arginase 1 primary antibody (1:200) and goat anti‐rabbit 594 secondary antibody (1:200). During confocal imaging, DAPI staining was used to locate the tumor centers for image acquisition. However, the DAPI channel was removed in the presented images for clarity, as the density of DAPI signal within the tumor made it difficult to clearly see staining of the markers (Arg‐1, CD8, Iba1).

The surface area to volume ratio of TAMs was assessed using Imaris Microscopy Image Analysis software (Bitplane, Zürich, Switzerland). Arg‐1 expression in TAMs was measured using ImageJ software (NIH, Bethesda, Maryland). Regions of interest (ROIs) were created based on the TAMs channel, and the integrated densities of the Arg‐1 signal within those ROIs were measured. To measure cytotoxic T cell infiltration, slices stained for CD8 were imaged in the tumor center. CD8^+^ T cell bodies were counted manually and normalized to area. Healthy microglia in the contralateral hemisphere were manually counted as well. Male and female (*n* = 5 each) tumor bearing mice were used for these analyses with three images analyzed and averaged per animal. Image analyses were performed blinded.

### 4.9 Survival study and evaluation of markers of disease progression

For longitudinal studies to evaluate survival and disease progression, brain tumor bearing mice were assigned randomly to a treatment group: control (*n* = 11), BLZ (*n* = 11), and DBLZ (*n* = 13). An intravenous dose of 100 mg/kg was administered via tail vein injection on day 10 after inoculation. Day 10 dosing was chosen to mimic clinical situations of delayed diagnosis. This time point corresponds to approximately halfway through disease progression when there is already a well‐formed tumor present. Mice were monitored daily and euthanized when presenting signs of morbidity (<75% of initial mass, immobility). Kyphosis scoring was performed daily by blinded graders based on the degree of spinal cord outward curvature as a marker of neurodegeneration.^45^, ^52^ A score between 0 and 3 was assigned, with 0 indicating healthy posture and 3 indicating severe curvature that does not resolve upon movement. To assess motor function as a marker of cancer progression,^48^ mice were recorded in open field experiments on days 12, 15, and 18 postinoculation. Mice were recorded for 10 min in an isolated environment and analyzed for distance traveled and velocity with EthoVision XT video tracking software (Noldus Information Tech, Inc., Leesburg, Virginia). Percent time spent rearing, stationary, and in horizontal movement were calculated based on video analysis by blinded graders.

### 4.10 Statistics

Statistics and plots were produced with Graphpad Prism v8.0 (San Diego, California). Significance between survival curves was calculated using the Mantel‐Cox test. Significance between kyphosis scores over time and markers of motor function were determined using two‐way analysis of variance (ANOVA). Comparisons between treatment groups in image analyses of the tumor immune environment were conducted with Student's *t*‐tests. All error bars presented in figures are mean ± SE.

## 5 CONCLUSIONS

We present a novel dendrimer conjugate for specific delivery of CSF‐1 receptor inhibitor BLZ945 to TAMs, upon systemic administration, in an orthotopic glioblastoma model. By conjugating BLZ945 to the dendrimer via pH and esterase sensitive linker, we enable triggered release of BLZ945 in intracellular and intratumor conditions. With targeted TAMs delivery, DBLZ significantly improved the reprogramming of TAMs in vivo and promoted tumor infiltration of cytotoxic T cells with a single intravenous dose administered halfway through disease progression. This immune repolarization translated to prolonged survival and improvements in motor and behavioral markers of disease progression and was achieved at a fraction of the doses used in prior free BLZ945 studies. These results suggest dendrimer delivery of TAMs targeted immunotherapies such as BLZ945 have significant potential for clinical translation to improve patient prognoses through localized manipulation of the tumor immune response.

## AUTHOR CONTRIBUTIONS

**Kevin Liaw:** Conceptualization; data curation; formal analysis; investigation; methodology; writing‐original draft; writing‐review and editing. **Rajasekhar Ramireddy:** Conceptualization; data curation; formal analysis; investigation; methodology. **Anjali Sharma:** Formal analysis; methodology; writing‐original draft; writing‐review and editing. **Jiangyu Li:** Formal analysis; investigation; methodology. **Michelle Chang:** Formal analysis; investigation. **Rishi Sharma:** Formal analysis; investigation. **Sebastian Salazar:** Formal analysis; investigation. **Sujatha Kannan:** Conceptualization; funding acquisition; resources. **Kannan Rangaramanujam:** Conceptualization; funding acquisition; project administration; resources; supervision; writing‐review and editing.

## CONFLICT OF INTEREST

The authors have awarded and pending patents relating to TAMs targeting ability of high surface hydroxyl dendrimers. R. M. K. and S. K. are cofounders and have financial interests in Ashvattha Therapeutics, Inc., Orpheris, Inc., and RiniSight, three startups undertaking clinical translation of the dendrimer drug delivery platform. RS currently works with Ashvattha Therapeutics and has share ownership. This work was performed before RS joined Ashvattha.

### PEER REVIEW

The peer review history for this article is available at https://publons.com/publon/10.1002/btm2.10205.

## Supporting information


**Appendix S1:** Supporting information

## Data Availability

Data are available on request from the authors.
