# Supplementary material for: Targeted systemic dendrimer delivery of CSF‐1R inhibitor to tumor‐associated macrophages improves outcomes in orthotopic glioblastoma
Source: Bioeng Transl Med. 2020 Dec 11;6(2):e10205. doi: 10.1002/btm2.10205 (PMC8126814; doi:10.1002/btm2.10205)
Supplement: Supplementary file 1 — Appendix S1: Supporting information [file BTM2-6-e10205-s001.docx]

**Dendrimer-mediated systemic delivery of CSF-1R inhibitor to tumor-associated macrophages improves outcomes in orthotopic glioblastoma**

Kevin Liaw,^1,2^ Rajsekhar Reddy,^1^ Anjali Sharma,^1^ Jiangyu Li,^3^ Michelle Chang,^1^ Rishi Sharma,^1^ Sebastian Salazar,^3^ Sujatha Kannan,^4^ Rangaramanujam M. Kannan^1,2,*^

**Supplementary Information.**

**
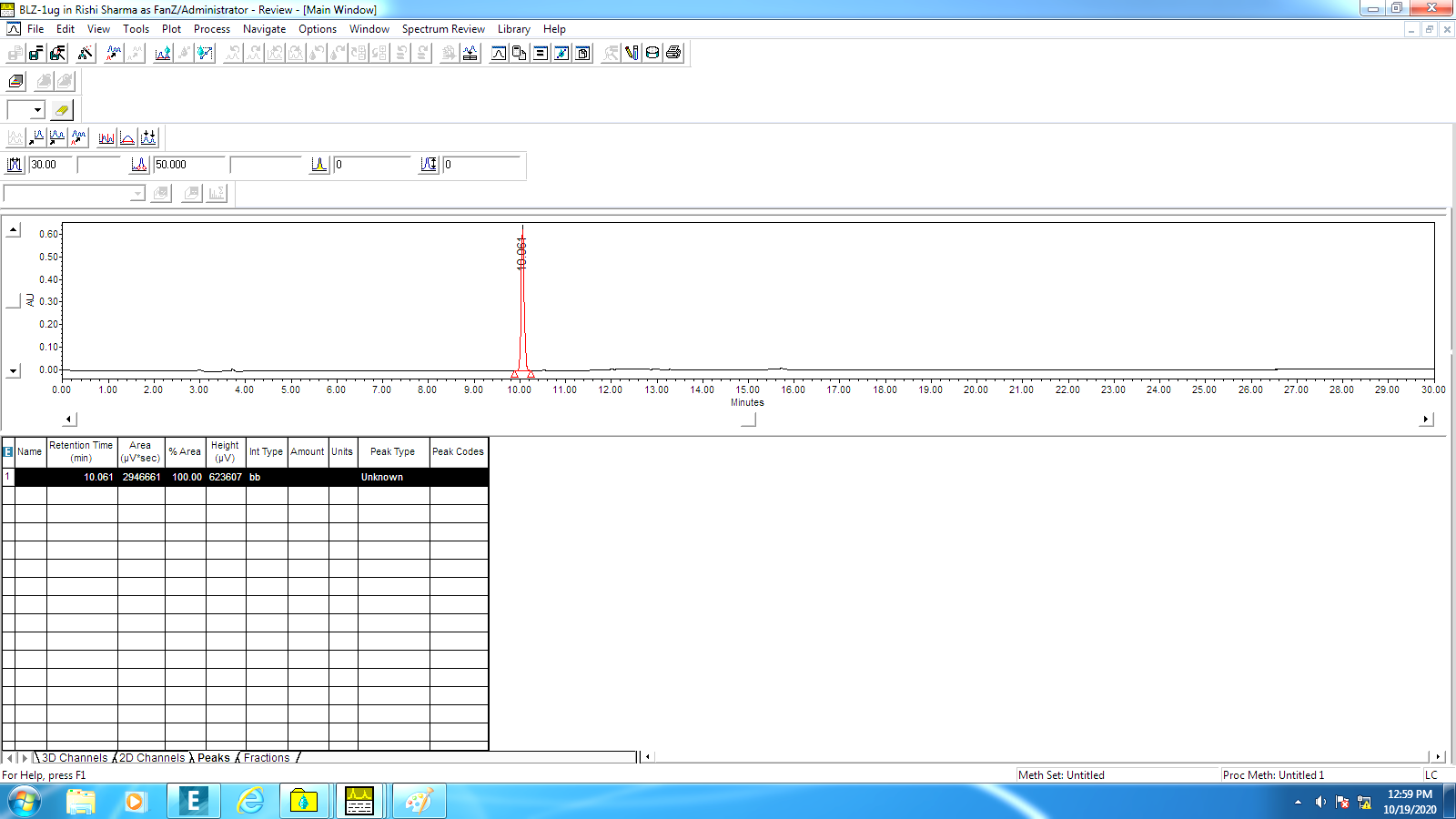
**

Purity: >99%

**Supplemental Figure 1.** HPLC trace of BLZ945 demonstrates >99% purity.


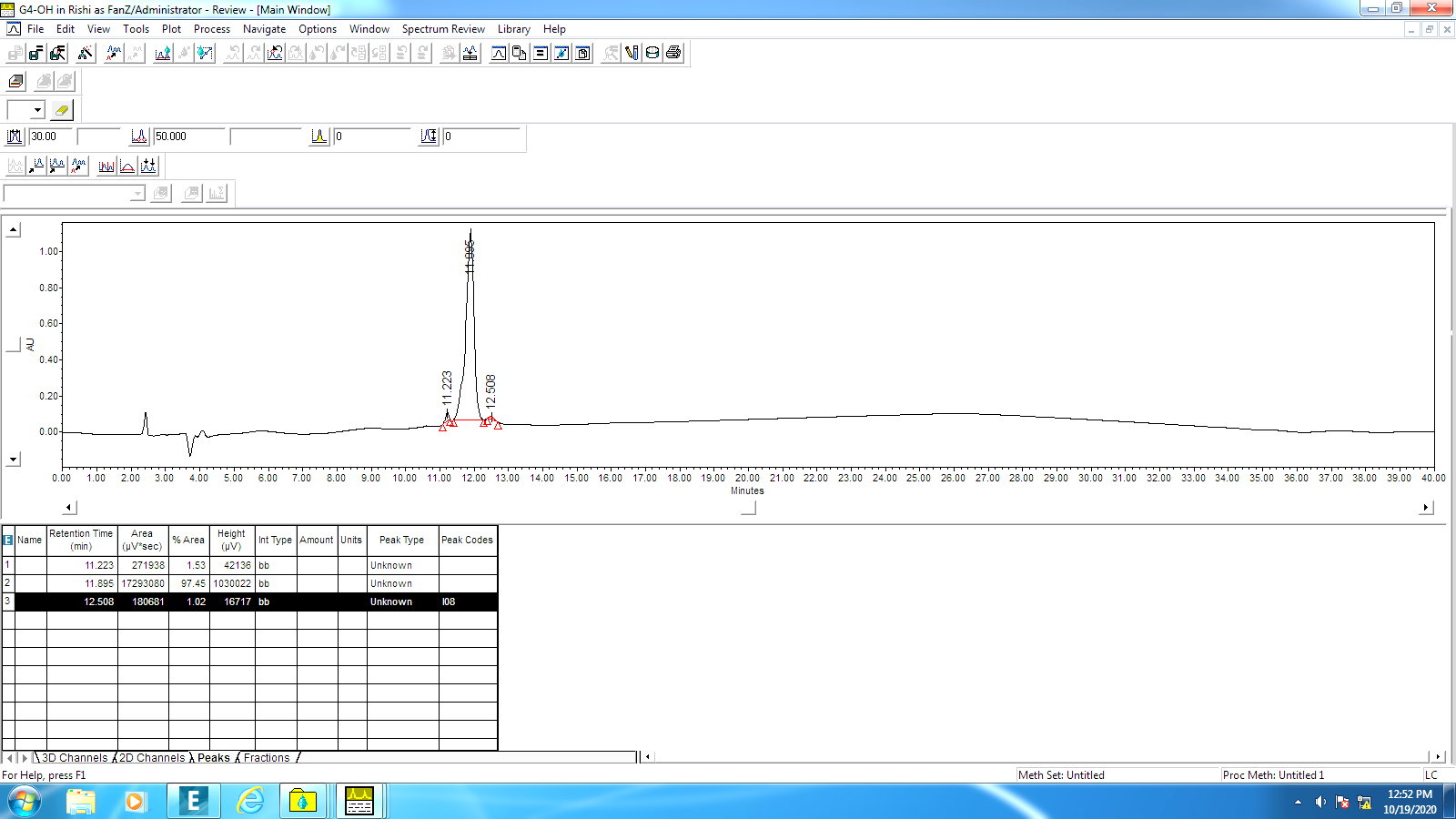

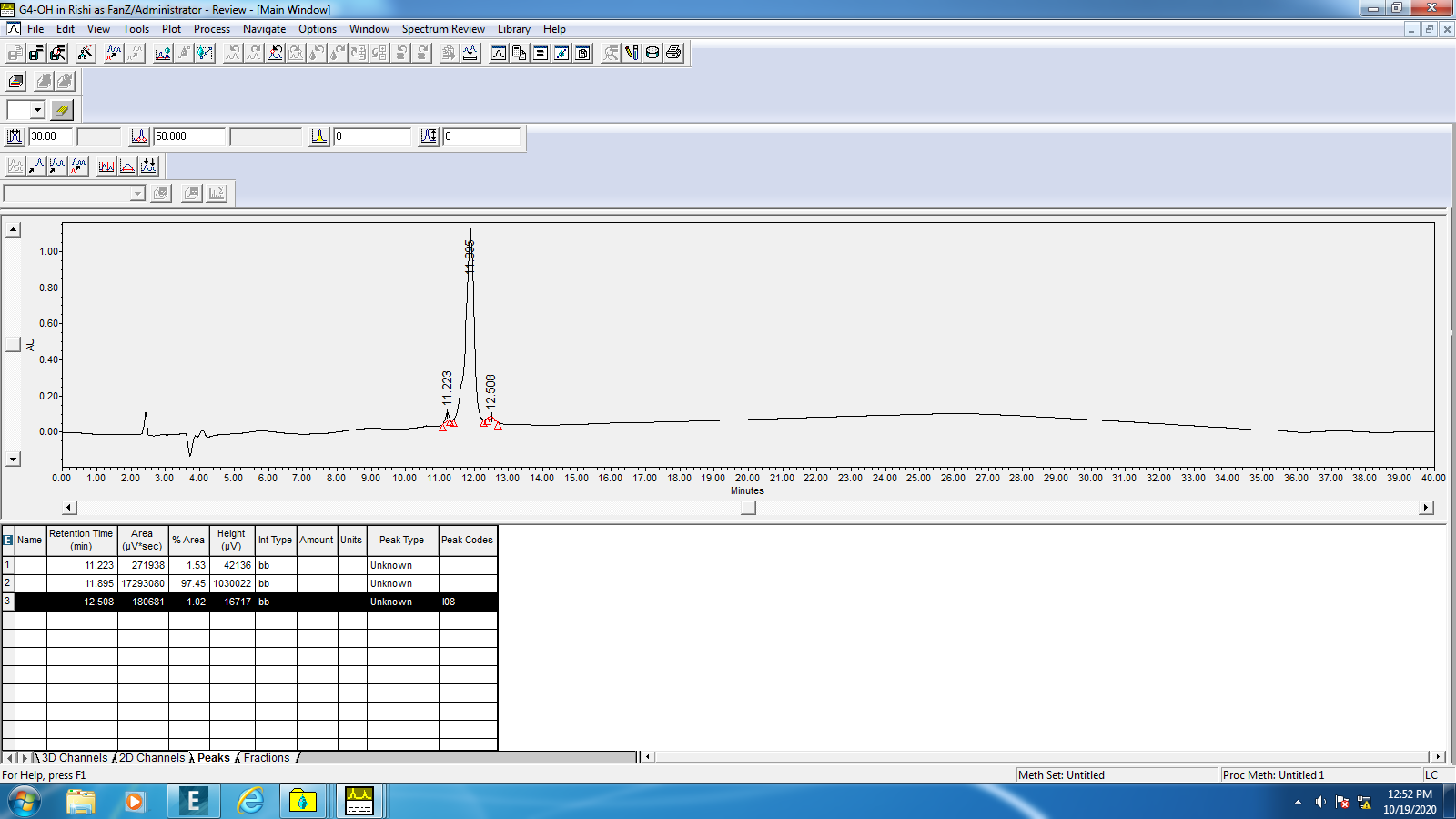


Purity: >97%

**Supplemental Figure 2.** HPLC trace of DBLZ demonstrates purity of >97% of the final conjugate.


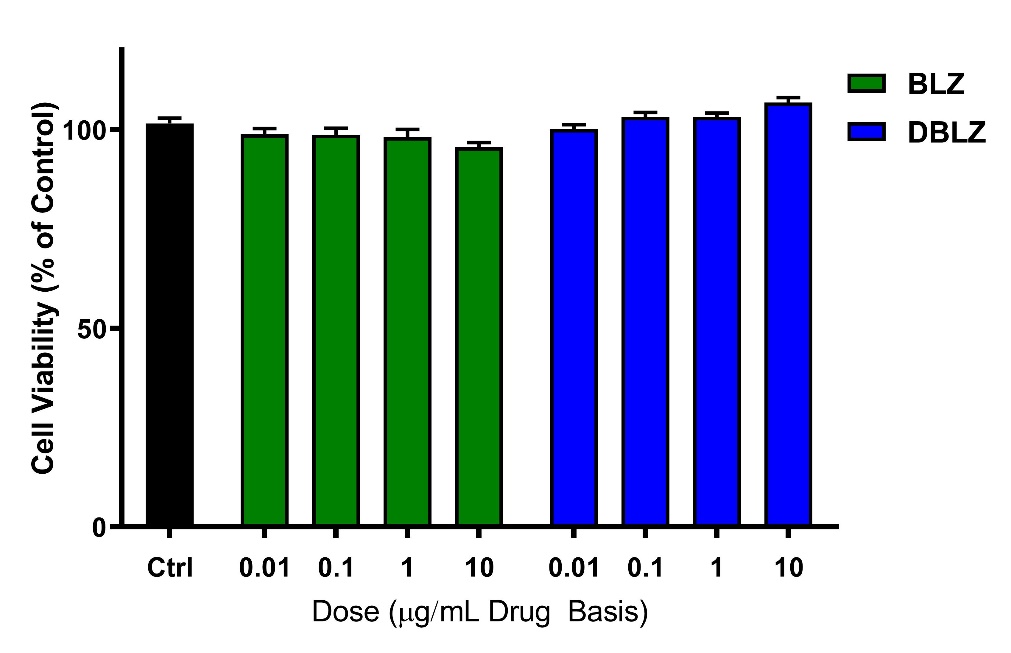


**Supplemental Figure 3.** Neither BLZ945 (BLZ) nor the dendrimer-BLZ945 conjugate (DBLZ) demonstrated cytotoxic effects in BV2 murine microglia after 24 hours of exposure.
